# Correction: Exploring diabetes status and social determinants of health influencing diabetes-related complications in a Northwestern community, Ontario, Canada: A mixed method study protocol

**DOI:** 10.1371/journal.pone.0308514

**Published:** 2024-08-02

**Authors:** Idevania G. Costa, Kristen McConnell, Kaitlin Adduono, Pilar Camargo-Plazas, Anna Koné

The second author’s name is spelled incorrectly. The correct name is Kristen McConnell. The correct citation is: Costa IG, McConnell K, Adduono K, Camargo-Plazas P, Koné A (2023) Exploring diabetes status and social determinants of health influencing diabetes-related complications in a Northwestern community, Ontario, Canada: A mixed method study protocol. PLOS ONE 18(9): e0273953. https://doi.org/10.1371/journal.pone.0273953.
