# Supplementary material for: Exploring diabetes status and social determinants of health influencing diabetes-related complications in a Northwestern community, Ontario, Canada: A mixed method study protocol
Source: PLoS One. 2023 Sep 28;18(9):e0273953. doi: 10.1371/journal.pone.0273953 (PMC10538706; doi:10.1371/journal.pone.0273953)
Supplement: Attachment — Submitted filename: Summary Responses to Reviewers March 21.pdf [file pone.0273953.s001.pdf]

March 21, 2023

To: Sana Sadiq Sheikh

Academic Editor
Plos One Journal

Re: PONE-D-22-20511 “Exploring diabetes status and social determinants of health influencing diabetes-related complications in a Northwestern Ontario - A mixed method study protocol”

Dear Dr. Sheikh,

We would like to thank you for allowing us to resubmit a revised copy of our manuscript and the reviewers for providing valuable comments and suggestions to improve it. We agree with most of the comments/suggestions made by the reviewers and have addressed them as outlined in the table below.

**Reviewer Comments**	**AUTHORS’ RESPONSE**<br>**NOTE:** Within the manuscript, all text changes are highlighted in **Yellow** .	**Line #**
**Reviewer #1**		
1. Please use same unique referencing format in whole the paper. For example, in the Introduction, line 5 you are suing two different formats. Same in the second page of Introduction, second paragraph line 7.	Thank you for this comment!	
2. Please justify why you are doing both quantitative and qualitative studies parallel?	Thank you for asking clarification. We have addressed this question in the methods section	101 to 109
3. For the quantitative study, please explain what the exclusion criteria are?	We have clarified it under study population section and added that: participants will be included if they meet the inclusion criteria…<br><br>We have also clarified our inclusion criteria: Those meeting these criteria but who are not active (i.e., not attending appointments) in the last 6 months of 2022 are excluded from the study.	154 to157<br><br>157 to 160
4. For the qualitative study, what are the exclusion criteria?	We have included the following: We will exclude women with gestational diabetes, people with pre-diabetes, a new diagnosis of diabetes, and a recent diagnosis of diabetes after an organ transplant, and people with mental illness with a diagnosis of diabetes who are not able to articulate their experience and journey of managing the disease	197 to 200
5. For the qualitative study, please explain what you will do to increase trustworthiness such as credibility, transferability,…(study rigor).	Table 3 was added to address this suggestion.	
6. Who will conduct the interviews?	Thank you for this comment. We have clarified it and added other missing information about the	181 to 184

	pilot testing of the quantitative questionnaire.<br>See the following:<br>The semi-structured interview guide will be pilot tested by the first (IGC) and third authors (KA) with three participants attending the CCDC and accepting participation in the study. Then the team will meet to make necessary amendments to the interview guide and the third author will continue to conduct the interviews	
**Reviewer #2**		
It was a pleasure to review and learn about your work which I believe is very relevant to the research community as well as population health. While I found this a fair read, I have the following comments and considerations to work on and address.	Thank you for your comments and suggestions! We really appreciate them.	
General comments<br>Pay attention to claims without appropriate interpretation of reference. Provide reference citations for assertions that are not yours.	Reviewed and addressed. We have added a few references (highlighted) to this updated version to address this comment.	
A few grammatical and syntactical errors are replete in your piece. Address them	The paper has been reviewed for grammatical and syntactical errors	
Ensure references are in alignment with PLONE author guidelines (including dois where necessary, date of access for URLs, etc)	References were reviewed and are now aligned with Plos One guideline	
Specific comments below:		
More context on the burden of DM on the local community in NWO compared to neighbouring areas would be necessary to understand and justify the research inquiry	References 9-11 were added to address this comment	
"While, the focus of most studies has been on pharmacological and technical approaches to manage diabetes," cite the studies that focus on pharmacological techniques.<br><br>I beg to disagree because a cursory online scan of studies on DM in NWO shows a few studies focusing on DM epidemiology and outcomes.	We have rephrased this statement and included more studies related to DM and NWO as stated in the next response	59 to 63
"To date, no published study has explored the impact of gender, race/ethnicity and social determinants of health on diabetes-related complications for individuals living in Northwestern Ontario".<br>As commented above, I disagree. Consider rephrasing as it is not totally true. There are a few studies albeit, localized or published over a decade. Often lazy scholars use the idea of 'no evidence' to rationalize study objectives when they failed to do a proper review of literature	We have adjusted and included more references to better reflect the state of evidence: To our knowledge, limited published studies exist related to diabetes in NWO communities [4,5, 8]. Also, the influence of social determinants of health on diabetes or diabetes-related complications, particularly the impact of gender, race/ethnicity for individuals living with diabetes in NWO have not been fully studied [13-15].	59 to 63

"Northwestern Ontario (NOW)" do you mean NWO here.	Correct! We have fixed it	
"However, there is evidence that Canadian population with diabetes who are living in remote and rural areas of Northwestern Ontario are facing lots of challenges in addressing social and economic determinants of health, particularly food insecurity and access to services and resources to support their diabetes management" Provide the evidence by citing the source!	We rephrased and added the sources:<br>Evidence exists that Canadians with diabetes living in remote and rural areas of NWO face many challenges in addressing the social and economic determinants of health, particularly food insecurity and access to services and resources to support their diabetes management [16-18]	64 and 67
"Population groups that have more difficulty to access, services, resources, and fresh and healthy food due to financial constraints and low-income households, include those with lower levels of education, Indigenous peoples, and those living in remote regions [14]. Therefore, nonpharmacological interventions that addresses the social determinants of health and enhance selfcare behaviours may lead to prevention of diabetes-related complications improve quality of life and decrease the economic burden of disease [15], especially for people with Type 2 diabetes in rural and remote areas of Northwestern Ontario."<br><br>This is a valid point but the delivery could be re-structured to show the linkages between socio-personal determinants of health and food insecurity which I think is the point you trying to drive home here.	This reviewer's comment was well taken. To address it we have made the following changes:<br>Social determinants of health represent an important factor, especially for population groups living in rural and remote areas. These populations usually experience food insecurity and difficulty in accessing healthcare services due to financial constraints and low income [15, 17, 18]. Therefore, it is crucial to consider this specific context when evaluating diabetes outcomes and the needs and concerns for people with diabetes in remote communities, which contribute to improve patients' outcomes and quality of life [14]. Such focus is particularly relevant and urgently needed for people with type 2 diabetes in rural and remote areas of NWO	69 to 75
"However, the relationships between social economic factor and individuals' attributes are not totally clear"<br><br>what does literature say about this that is not totally clear? Include it, analysing for the gaps in literature and how your proposal will help bridge that gap. Don't just provide vague and generic statements.	Thank you for your comment. We have made the following changes:<br>While a few studies address social determinants of health for people living with diabetes in rural and remote NWO [7, 9, 10, 20], the relationships between socioeconomic factors and individual attributes such as gender and race/ethnicity have not been explicitly studied in the context of a population living in NWO, Canada	79 to 82
"Thus, in response to this gap, this research study will firstly use a cross-sectional design to identify the prevalence of diabetes complications, the internal and external factors affecting diabetes' outcomes in a Northwestern Community. Secondly, it will employ a critical pedagogy approach to explore the needs, motivations and struggles individuals with diabetes face to self-manage the disease on a	Agreed and moved to methods section.	101 to 109

daily basis." This should be for the methods section. Move it.		
Methods<br>"parallel mixed-method approach" it will be a good place to explain parallel mixed-method approach and why it best fits to answer your research questions.	Thank you for asking clarification. We have addressed these questions in the methods section by adding the following:<br>A mixed-method approach was chosen in order to combine the benefits of both quantitative and qualitative methods by using numbers and narratives that will better reflect individuals' experiences; and hence, help us achieve the four research objectives [21]. In addition, collecting diverse types of data creates data triangulation and helps us to have greater insights on the diabetes status, care, needs, and challenges faced by individuals in NWO. It also provides more valid and stronger inferences than a sole method would achieve [22].	101 to 109
The region of NWO has a significant amount of Indigenous communities of whom have higher inequalities socially, and clinically.<br><br>I didn't see how your proposal is designed to address Indigenous health from a community-led/engaged perspective. Indigenous peoples have higher rates of DM than non-Indigenous peoples. What engagement strategies, protocols and pursuits are in place to address inequalities in for this group of people?	Thank you for your concern. Our study does not aim at proposing strategies or protocols to address inequality. To avoid this misunderstanding, we have rewarded the last sentence of the paragraph before objectives to:<br>"Such knowledge may help support the institutions providing diabetes care in NWO to develop strategies to improve diabetes and diabetes-related outcomes, enhance self-management skills, wellness, health accessibility, and equitable care among people living with diabetes in NWO.<br><br>Hope it makes sense now.	88-91
I need more information on how the findings from this study would benefit patients with DM with regard to self-care, self-reliance, health promotion, and rehospitalization. Your focus is more on policy and population health but less on the individual scale.	Correct! Our focus is on collecting information about the social determinant's health affecting diabetes outcomes along with participants' perceptions of their needs, struggles and motivations to manage diabetes. Patients will indirectly benefit from this research findings as it contributes to inform policy and the development of strategies, such as self-care program for people living with diabetes in NWO.<br><br>We have reworded "Research significance. Section" to help clarify it.	235-249
Have included how your study will not add to increase health inequalities in other areas of chronic disease care and will address equity, diversity and inclusion of participants.	Thank you for your question.<br>As the study is only focusing on diabetes, we understand that more work is need for other chronic conditions, given the increasing burden of multimorbidity. However, this is a step in the right direction which may inform other areas, and we will be considering the coexistence of	

	other conditions in our analyses and patients' narratives.<br>While all eligible people are included regardless of their characteristics, there is a limitation to address specific aspects of equity and inclusion as, for example we do not have a targeted approach to focus on indigenous people.	
Comment on knowledge translation considerations as well. How will data findings be made available to relevant stakeholders	We have a section related to knowledge dissemination and added the following: A knowledge translation plan informed by Farkas et al. [29] and Lavis et al. [30] has supported us to develop multiple strategies to disseminate research' findings to a diversity of stakeholders. (cont.)	251 to 265

Thank you for the opportunity to review our manuscript; we believe that the reviewers' thorough review and thoughtful comments have vastly contributed to improve its quality.

Sincerely,

The Authors
